# Integrative taxonomy reveals a new species of the genus *Burmoniscus* (Isopoda, Philosciidae) from the Xuefeng Mountains, China

**DOI:** 10.3897/zookeys.1055.66879

**Published:** 2021-08-10

**Authors:** Xue-Gang Zeng, Jin Wang, Jing-Bo Yang, Wei-Chun Li

**Affiliations:** 1 College of Agronomy, Jiangxi Agricultural University, Nanchang 330045, China Jiangxi Agricultural University Nanchang China

**Keywords:** Molecular, morphology, new species, Oniscidea

## Abstract

Three species of the genus *Burmoniscus* are identified from the Xuefeng Mountains, central China, by integrating morphological and molecular approaches. *Burmoniscuschuanyanensis* Li, **sp. nov.** is described. Morphological photographs of the new species are provided.

## Introduction

The genus *Burmoniscus* was erected by Collinge (1914) to allocate *B.moulmeinus* [a junior synonym of *B.coecus* (Budde-Lund, 1895)]. The genus is mainly defined by the pereonites with sulcus marginalis and gland pores, *noduli laterales* on pereonites 2 and 4 shifted from lateral margins, and epimera of pleon reduced and adpressed; the head with supra-antennal line and no frontal line, mandibles with molar penicil reduced to a single plumose seta, outer lobe of maxillule with 4+6 (5 apically cleft) teeth, inner lobe with two unequal penicils and a small posterior point; uropod protopodite grooved on outer margin; insertion of endo- and exopodite at the same level; pleopod exopodites without respiratory areas (Taiti and Ferrara 1986). To date, the genus comprises 73 species distributed worldwide, of which the Oriental, Afrotropical, and Australian regions have a greater diversity (Taiti and Ferrara 1986; Kim and Kwon 2002; Kwon and Kim 2002; Schmalfuss 2003; Taiti and Ferrara 2004; Jeon and Kwon 2009; Karasawa 2016).

Prior to this study, 21 *Burmoniscus* species have been recorded from China (Kwon and Jeon 1993; Kwon and Taiti 1993; Dai and Cai 1998; Nunomura and Xie 2000). Among them, most members were reported from southwestern and eastern China, but the species of central China are poorly known. Moreover, no record of the genus is known from the Xuefeng Mountains, the largest mountain range of Hunan Province, central China.

In general, the taxonomy of terrestrial isopods using multiple methods has proved to be an efficient strategy in delimiting species (Karasawa 2016; Zimmermann et al. 2018a, b; Bedek et al. 2019). In this context, we studied the specimens collected from the region of the Xuefeng Mountains by integrating morphological and molecular characters. Three *Burmoniscus* species are identified, including one new species.

## Materials and methods

### Morphological study

Specimens were collected by hand with tweezers, fixed in absolute ethanol, and stored at −20 °C in a freezer. The appendages were dissected and mounted on micro-preparations in neutral balsam mounting medium. Photographs were taken with a digital camera Zeiss AxioCam Icc 5 attached to a digital microscope Zeiss Stereo Discovery V12. The line drawings were drawn by the GNU Image Manipulation Program (Montesanto 2015). All the specimens are deposited in the Museum, Jiangxi Agricultural University, Nanchang, China (**JXAUM**).

### DNA extraction, amplification, and sequencing

Genomic DNA was extracted from pereonites of the samples (Table 1) using the TaKaRa MiniBEST Universal Genomic DNA Extraction Kit. Parts of the mitochondrial cytochrome c oxidase subunit I (COI) were amplified using the primers LCO1490 and HCO2198 (Folmer et al. 1994). PCR amplifications were performed with an initial denaturation at 95 °C for 3 min, followed by 35 cycles of 30 sec at 94 °C, 30 sec at 50 °C, and 1 min 72 °C, with a final extension at 72 °C for 10 min. The PCR products were sequenced by using an ABI3730XL DNA Analyzer (Applied Biosystems). All sequences were deposited in DDBJ (DNA Data Bank of Japan), with accession numbers listed in Table 1.

**Table 1.** Samples of COI genes used in this study.

Species	DNA number	Locality	Accession number
*B.chuanyanensis* Li, sp. nov.	CYS2007	China, Hunan, Chuanyanshan	LC617864
	CYS2008	China, Hunan, Chuanyanshan	LC617865
	CYS2009	China, Hunan, Chuanyanshan	LC617866
	CYS2010	China, Hunan, Chuanyanshan	LC617867
	CYS2011	China, Hunan, Chuanyanshan	LC617868
*B.kathmandius* Schmalfuss, 1983	LCS2022	China, Hunan, Lingcuishan	LC617869
	LCS2023	China, Hunan, Lingcuishan	LC617870
	LCS2024	China, Hunan, Lingcuishan	LC617871
	LCS2027	China, Hunan, Lingcuishan	LC617872
		Japan, Okinoerabujima	LC075193
*B.mauritiensis* (Taiti & Ferrara, 1983)	LCS2028	China, Hunan, Lingcuishan	LC617873
	LCS2029	China, Hunan, Lingcuishan	LC617874
*B.dasystylus* Nunomura, 2003		Japan, Wakayama, Shirahama	AB626154
*Ligidiumryukyuense* Nunomura, 1983		Japan, Amami-Oshima	AB626261
*Ligiaexotica* Roux, 1828		China, Guangxi, Beihai	KC762932

### Sequence processing and phylogenetic analysis

A total of 11 mitochondrial COI sequences were obtained. In addition, two *Burmoniscus* sequences and another two sequences representing the outgroups were downloaded from NCBI for phylogenetic analysis (Table 1). The estimation of sequence divergences was conducted using the Kimura two-parameter model (K2P) method in MEGA X including all sites, with the pairwise deletion option (Kumar et al. 2018). Sequences were aligned in MAFFT 7 (Katoh and Standley 2013). The best-fit partition model was select using the BIC criterion with ModelFinder (Kalyaanamoorthy et al. 2017) on the platform of PhyloSuite (Zhang et al. 2020). ModelFinder identified the HKY+G+F/TPM2+R4+F model as the best fit for Bayesian (BI) analysis and maximum likelihood (ML) analyses. The BI analysis was conducted in MrBayes v. 3.2.6 (Ronquist et al. 2012). Four chains of Markov chain Monte Carlo (MCMC) were run simultaneously for a total of 10,000 generations; the sampling frequency was set to 100, and the number of runs and burnin fraction with 2 and 0.25, respectively. The ML trees were constructed based on the above best models with 1000 bootstrap replicates using IQ-TREE 2 (Minh et al. 2020).

## Results

### Species delimitation based on COI and morphological characters

**Figure 1.**
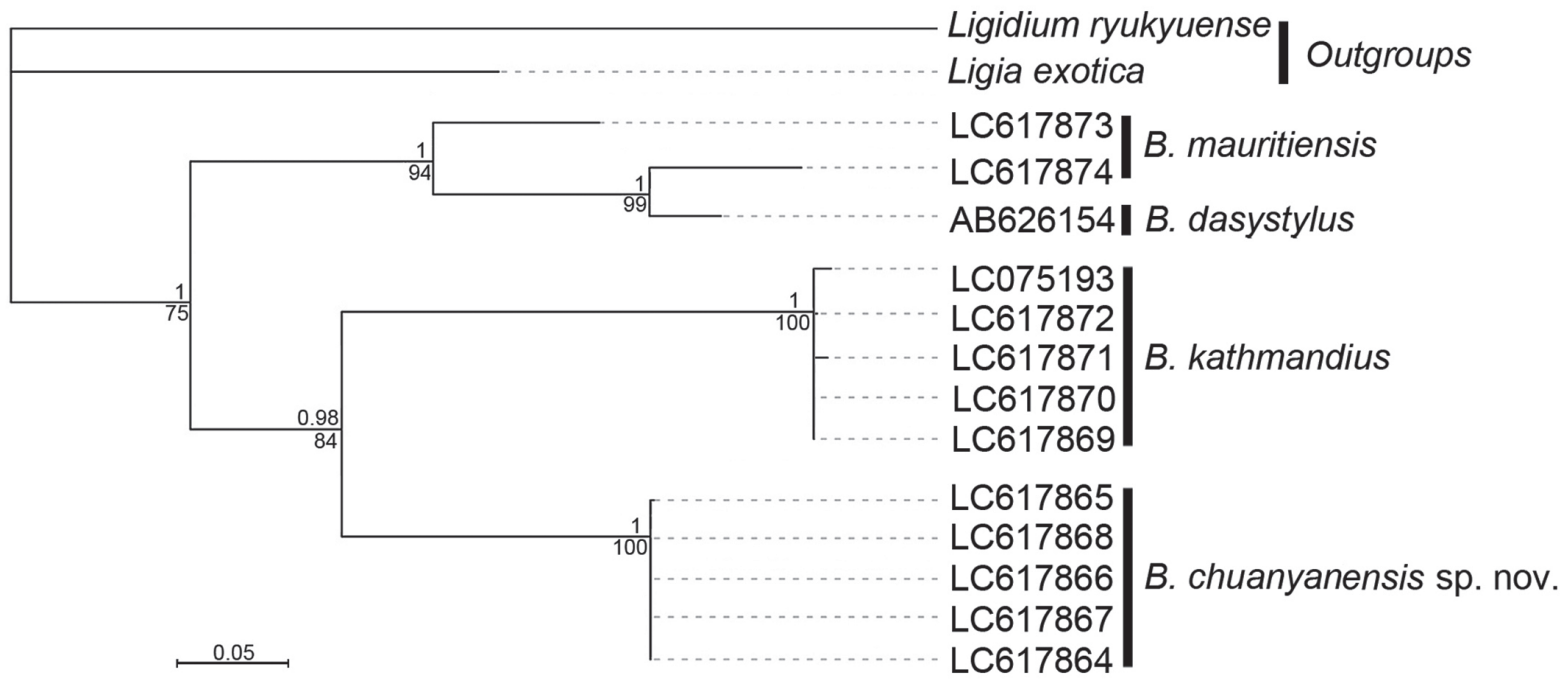
Maximum likelihood (ML) tree of the COI dataset with best ModelFinder model applied. Values below the nodes represent bootstrap support (BS) values from 1000 replicates, and values above the nodes represent the posterior probability (PP) of the Bayesian analysis of the same dataset.

**Figure 2.**
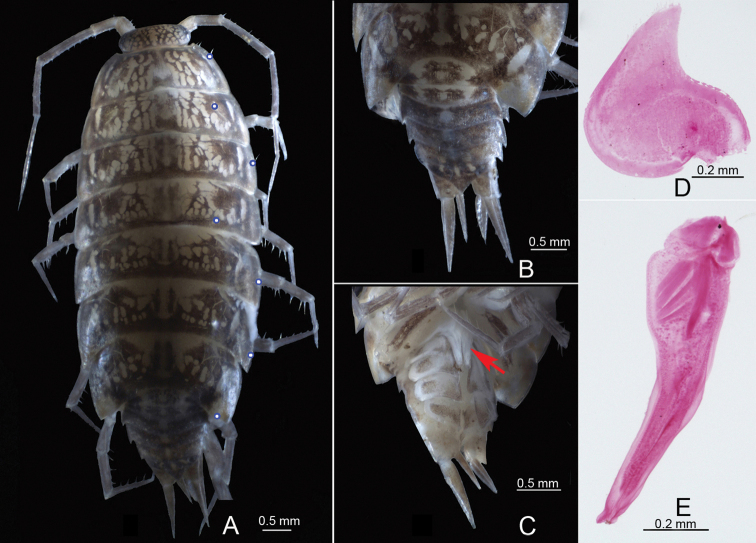
*Burmoniscuschuanyanensis* Li, sp. nov., paratype, male **A** habitus in dorsal view; blue circles showing insertion of *noduli laterales***B** pleon in dorsal view **C** pleon in ventral view **D** pleopod 1 exopodite **E** pleopod 1 endopodite.

**Figure 3.**
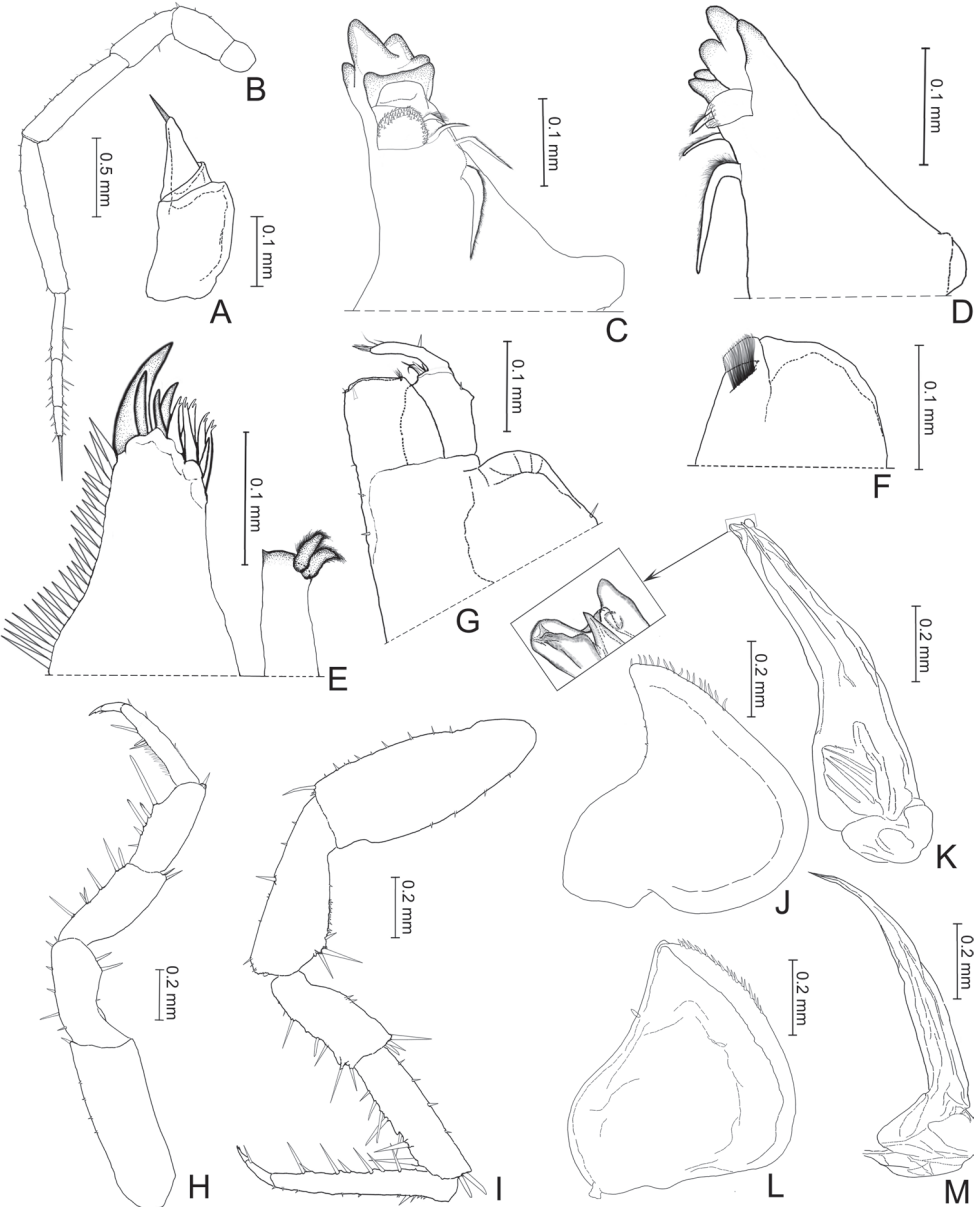
*Burmoniscuschuanyanensis* Li, sp. nov., holotype **A** antennule **B** antenna **C** left mandible **D** right mandible **E** maxillule **F** maxilla **G** maxilliped **H** pereopod 1 **I** pereopod 7 **J** pleopod 1 exopodite **K** pleopod 1 endopodite **L** pleopod 2 exopodite **M** pleopod 2 endopodite.

**Figure 4.**
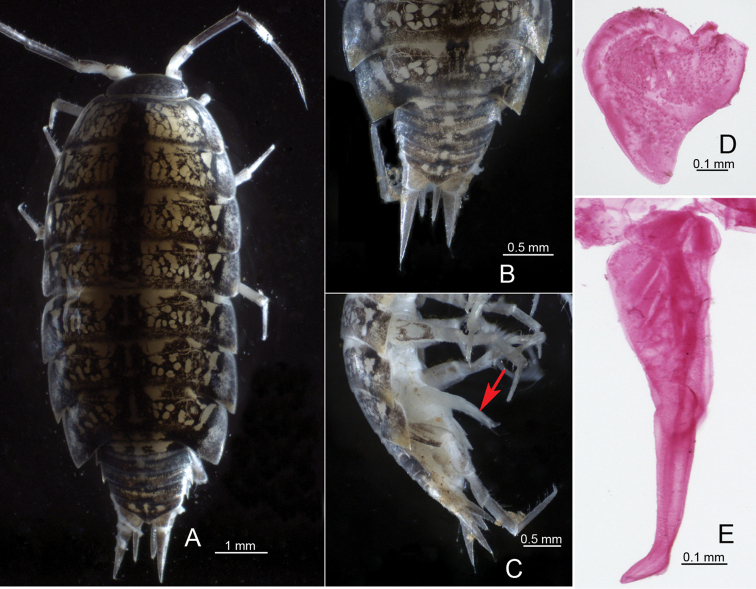
*Burmoniscusmauritiensis* (Taiti & Ferrara, 1983), male **A** habitus in dorsal view **B** telson in dorsal view **C** pleon in lateral view **D** pleopod 1 exopodite **E** pleopod 1 endopodite.

**Figure 5.**
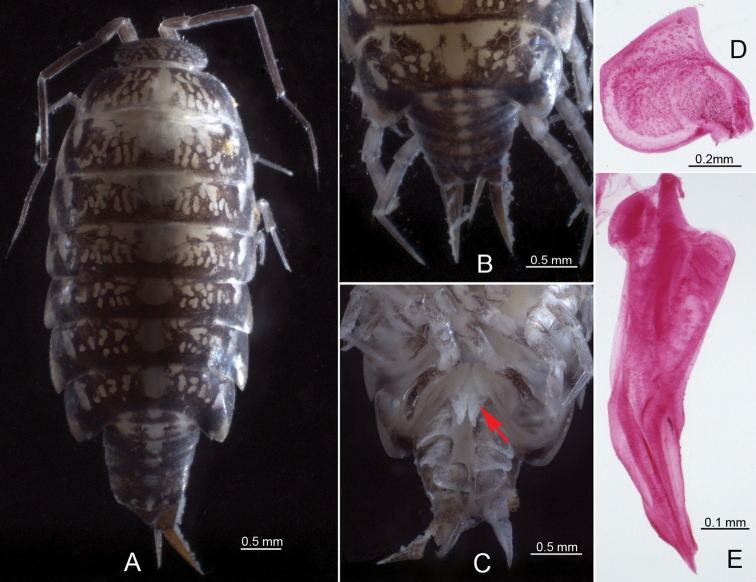
*Burmoniscuskathmandius* (Schmalfuss, 1983), male **A** habitus in dorsal view **B** telson in dorsal view **C** pleon in ventral view **D** pleopod 1 exopodite **E** pleopod 1 endopodite.

All results are based on the pairwise analysis of the 15 sequences (Table 1). In this study, we acquired 11 COI sequences representing three *Burmoniscus* species. All the new sequences are 658 bp in length and were deposited in DDBJ (accession no. LC617864 to LC617874). The extremes of inter- and intraspecific distances are presented in Table 2. Both BI and ML analyses represent the same topologies and revealed three clades with high support values (Fig. 1). Three molecular operational taxonomic units (MOTUs) from the Xuefeng Mountains were detected in the molecular analyses, which are consistent with the results of morphological taxonomy (Figs 2–5) for *B.chuanyanensis* Li, sp. nov., *B.mauritiensis* (Taiti & Ferrara, 1983), and *B.kathmandius* Schmalfuss, 1983.

**Table 2.** Pairwise genetic divergence (K2P-distance) among *Burmoniscus* species (bold) and the outgroup using COI gene sequences.

	1	2	3	4	5
1 ***B.mauritiensis***	**0.195**				
2 ***B.dasystylus***	**0.096**				
3 ***B.kathmandius***	**0.270**	**0.297**	**0.014**		
4 ***B.chuanyanensis***	**0.249**	**0.258**	**0.237**	**0.002**	
5 *Ligiaexotica*	0.335	0.358	0.382	0.336	
6 *Ligidiumryukyuense*	0.303	0.318	0.301	0.298	0.334

### Taxonomic account

#### Burmoniscus chuanyanensis

Taxon classificationAnimaliaIsopodaPhilosciidae

Li sp. nov.

CF5DAD2E-4D94-5EC4-8987-7AD75C3BC57D

http://zoobank.org/58335AE6-9B0B-4668-B726-9B4328A20DD9

Figures 2A–E , 3A–M

##### Type material.

***Holotype*.** China, male, Hunan Province, Xuefeng Mountains, Chuanyanshan National Forest Park (27°42'N, 110°34'E), alt. 391 m, 14 July 2019, Wei-Chun Li and Jing-Bo Yang leg., Prep. slide no. L2007–2009, DNA no. CYS2007–2011.

***Paratypes*.** One male, 23 females, same collection data as holotype.

##### Diagnosis.

Male pleopod 1 endopodite with distal portion bearing bilobated apical tip with one subapical spine-like projection between lobes.

##### Description.

Body length of male 7.5 mm, of females 3.5–9.0 mm. Body elliptic. Color brown with pale muscle spots on dorsal surface in preserved specimens with ethanol (Fig. 2A). Telson triangular, lateral margin slightly concave, apex acute (Fig. 2B). Uropod protopodite almost twice as long as wide, exopodite approximately twice as long as endopodite (Fig. 2B, C).

***Antennule*.** Composed of three articles, distal article bearing apical set of small aesthetasc (Fig. 3A). *Antenna*. Flagellum slightly shorter than fifth article of peduncle, scattered with tiny setae, length ratio of flagellar articles 6: 4: 3, third article ending with slender apical organ, slightly longer than third article (Fig. 3B). *Mandibles*. Left mandible bearing 2+1 penicils, right mandible with 1+1 penicils, molar process consisting of single plumose seta (Fig. 3C, D). *Maxillule*. Inner lobe with two stout penicils and small posterior point; outer lobe with 4 + 6 (5 apically cleft) teeth (Fig. 3E). *Maxilla*. Distally divided into two lobes with blunted rounded margins; inner lobe covered with thick setae (Fig. 3F). *Maxilliped*. Endite nearly rectangular, subapically with penicil; palp two-segmented (Fig. 3G).

***Pereopods*** (Fig. 3H, I). Pereopod 1 and pereopod 7 without sexual dimorphism.

***Pleopods, sexual differentiation*.** Male pleopod 1 exopodite heart-shaped, outer margin sinuous, inner margin broadly rounded, distal portion triangular bearing one row of small setae on inner margin (Fig. 3J); endopodite with broad basal part, narrowed towards apical tip, apical tip bilobed, subapical part bearing one spine-like projection on middle (Fig. 3K). Pleopod 2 exopodite slightly concave on outer margin, inner margin slightly convex and covered one line of small setae at distal portion (Fig. 3L); endopodite longer than exopodite, with distal article long and narrowed apically (Fig. 3M).

##### Etymology.

The new species is named after the national forest park where the type specimens were collected.

#### Burmoniscus mauritiensis

Taxon classificationAnimaliaIsopodaPhilosciidae

(Taiti & Ferrara, 1983)

C8501CF5-0B60-5B0F-8E84-09BCA75219E2

Figure 4A–E

Rennelloscia mauritiensis Taiti & Ferrara, 1983: 203, fig. 2.
Burmoniscus mauritiensis (Taiti & Ferrara, 1983): Taiti and Ferrara 1986: 187; Kwon and Taiti 1993: 17.

##### Examined specimens.

Three males, seven females, China, Hunan Province, Xuefeng Mountains, Lingcuishan Park (27°54'N, 110°34'E), 15 July 2019, alt. 214 m, Wei-Chun Li and Jing-Bo Yang leg., slide prep. no. L2016–2018, DNA no. LCS2028–2029; 13 males, 85 females, China, Jiangxi Province, Nanchang County, Liantang (28°33'N, 115°56'E), 3 January 2012, Wei-Chun Li leg., slide prep. no. L17225–17230.

##### Distribution.

China (Guangxi, Hong Kong, Hunan, Jiangxi, Nanjing, Sichuan); Hawaiian Islands; Korea; Mauritius.

##### Remarks.

In the examined specimens of this species, the body length of males were 4.0–7.0 mm, and of females 4.5–7.0 mm. Taiti and Ferrara (1983) have adequately described and illustrated this species. By morphology, it is easily distinguished by the unique characters of the male pleopod 1 endopodite, which is distinctively bent outwards near the apical area and densely covered with denticles (see Taiti and Ferrara 1983: fig. 2). Furthermore, the morphological characters of this species are identical to those of *B.dasystylus* Nunomura, 2003 (see also Nunomura 2003a: fig. 5).

Karasawa (2016) provided a COI sequence (accession no. AB626154) of *B.dasystylus*. In this context, we estimated the COI sequence divergences of *B.mauritiensis* and *B.dasystylus* based on our data (accession no. LC617873, LC617874) and the published sequence (accession no. AB626154). The result shows that their minimum interspecific distance is much smaller than the maximum intraspecific distance of *B.mauritiensis* (Table 2). In the phylogenetic analysis, they were also represented by a well-supported clade (Fig. 1). Thus, according to the original descriptions and molecular analyses, *B.dasystylus* is most probably a junior synonym of *B.mauritiensis* and that a re-examination of the type material is necessary to confirm the synonymy.

#### Burmoniscus kathmandius

Taxon classificationAnimaliaIsopodaPhilosciidae

(Schmalfuss, 1983)

FACB2616-B240-5743-9EDB-81DC434EEC10

Figure 5A–E

Rennelloscia kathmandia Schmalfuss, 1983: 379, figs 5, 6, 9, 22, 23. Type locality: Nepal, Kathmandu-Balaju.
Burmoniscus kathmandius (Schmalfuss, 1983): Taiti and Ferrara 1986: 187; Karasawa 2016: 1.
Burmoniscus aokii (Nunomura, 1986): Karasawa 2016: 1, figs 3A, 5–8A, 10–14A, 16–17A (junior synonym of B.kathmandius).
Burmoniscus boninensis (Nunomura, 1986): Karasawa 2016: 1, figs 3A, 5–8B, 10–14B, 16, 17A (junior synonym of B.kathmandius).
Burmoniscus daitoensis (Nunomura, 1986): Karasawa 2016: 1, figs 3B, 5C, 6B, 7, 8C, 10–14C, 16, 17B (junior synonym of B.kathmandius).
Burmoniscus hachijoensis Nunomura, 2007: 25, fig. 5; Karasawa 2016: 1, figs 3C, 5D, 6C, 7, 8D, 10–14D, 16, 17C (junior synonym of B.kathmandius).
Burmoniscus japonicus (Nunomura, 1986): Karasawa 2016: 1, figs. 3D, 5E, 6D, 7, 8E, 10–14E, 16, 17D (junior synonym of B.kathmandius).
Burmoniscus kagoshimaensis Nunomura, 2003a: 31, fig. 4; Karasawa 2016: 1, figs 3E, 5F, 6E, 7, 8F, 10–14F, 16, 17E (junior synonym of B.kathmandius).
Burmoniscus murotoensis (Nunomura, 1986): Karasawa 2016: 1, figs 3F, 5G, 6F, 7, 8G, 10–14G, 16, 17F (junior synonym of B.kathmandius).
Burmoniscus okinawaensis (Nunomura, 1986): Karasawa 2016: 1, figs 3G, 5H, 6G, 7, 8H, 10–14H, 16, 17G (junior synonym of B.kathmandius).
Burmoniscus shibatai (Nunomura, 1986): Karasawa 2016: 1, figs 3H, 5I, 6H, 7, 8I, 10–14I, 16, 17H (junior synonym of B.kathmandius).
Burmoniscus tanabensis Nunomura, 2003b: 16, fig. 3; Karasawa 2016: 1, figs. 3I, 5J, 6I, 7, 8J, 10–14J, 16, 17I (junior synonym of B.kathmandius).
Burmoniscus watanabei (Nunomura, 1986): Karasawa, 2016: 1, figs 3J, 5K, 6J, 7, 8K, 10–14K, 16, 17J (junior synonym of B.kathmandius).

##### Examined specimens.

Seven males, 59 females, China, Hunan Province, Xuefeng Mountains, Lingcuishan Park (27°54'N 110°34'E), 15 July 2019, alt. 214 m, slide prep. no. L2013–2015, DNA no. LCS2022–2024 and LCS2027.

##### Distribution.

China (Guangdong, Guangxi, Hong Kong, Hunan, Taiwan); Japan; Nepal; Hawaiian Islands.

##### Remarks.

This species has the body length of males 3.5–5.0 mm, and of females 4.5–7.5 mm. Karasawa (2016) redescribed and illustrated the morphological characters in detail. It can be distinguished by the characters of the male pleopod 1 endopodite (Fig. 5E); however, morphological variations of the diagnostic characters have caused some taxonomic misinterpretation. Karasawa (2016) clarified the confusion through a combined morphological and molecular approach, and 11 species were considered to be junior synonyms of *B.kathmandius*. The present work identified the specimens from the Xuefeng Mountains by integrating morphological characters (Fig. 5A–E) and a phylogenetic analysis (Fig. 1).

## Discussion

In the original species descriptions, the male pleopod 1 generally provides the most significant diagnostic character, especially the apical portion of the endopodite, which shows a considerable difference in shape among the species. However, it is difficult to distinguish the interspecific morphological differences and intraspecific morphological variations in the closely related congeners. Thus, the assistance of the other methods is necessary to solve morphological problems (Karasawa 2016; Zimmermann et al. 2018a, b; Bedek et al. 2019). In this context, we examined the *Burmoniscus* species from the Xuefeng Mountains, central China, for the first time using an integrative taxonomical approach. Our results showed that the male apical structure of the pleopod 1 endopodite is a reliable diagnostic character. To delimit the species more precisely, we supplemented the molecular analyses and revealed three species, including a new species. Certainly, more species in this genus will be discovered in China with further sampling.

## Supplementary Material

XML Treatment for Burmoniscus chuanyanensis

XML Treatment for Burmoniscus mauritiensis

XML Treatment for Burmoniscus kathmandius
